# Revealing the crosstalk between LOX^+^ fibroblast and M2 macrophage in gastric cancer by single-cell sequencing

**DOI:** 10.1186/s12885-024-12861-y

**Published:** 2024-09-09

**Authors:** Dapeng Chen, Wen Tong, Bing Ang, Yi Bai, Wenhui Dong, Xiyue Deng, Chunjiong Wang, Yamin Zhang

**Affiliations:** 1 Tianjin First Central Hospital Clinic Institute, Tianjin Medical University, Tianjin, 300192 China; 2 Oncology Department, Tianjin First Central Hospital, School of Medicine, Nankai University, Tianjin, 300192 China; 3 Department of Hepatobiliary and Pancreatic Surgery, Tianjin First Central Hospital, School of Medicine, Nankai University, Tianjin, 300192 China; 4 Department of Physiology and Pathophysiology, Tianjin Medical University, Tianjin, China

**Keywords:** Gastric cancer, Tumor microenvironment, Prognosis, Fibroblast, Macrophage

## Abstract

**Background/Aims:**

Gastric cancer (GC) ranks among the prevalent types of cancer, and its progression is influenced by the tumor microenvironment (TME). A comprehensive comprehension of the TME associated with GC has the potential to unveil therapeutic targets of significance.

**Methods:**

The complexity and heterogeneity of TME interactions were revealed through our investigation using an integrated analysis of single-cell and bulk-tissue sequencing data.

**Results:**

We constructed a single-cell transcriptomic atlas of 150,913 cells isolated from GC patients. Our analysis revealed the intricate nature and heterogeneity of the GC TME and the metabolic properties of major cell types. Furthermore, two cell subtypes, LOX^+^ Fibroblasts and M2 Macrophages, were enriched in tumor tissue and related to the outcome of GC patients. In addition, LOX^+^ Fibroblasts were significantly associated with M2 macrophages. immunofluorescence double labeling indicated LOX^+^ Fibroblasts and M2 Macrophages were tightly localized in GC tissue. The two cell subpopulations strongly interacted in a hypoxic microenvironment, yielding an immunosuppressive phenotype. Our findings further suggest that LOX^+^ Fibroblasts may act as a trigger for inducing the differentiation of monocytes into M2 Macrophages via the IL6-IL6R signaling pathway.

**Conclusions:**

Our study revealed the intricate and interdependent communication network between the fibroblast and macrophage subpopulations, which could offer valuable insights for targeted manipulation of the tumor microenvironment.

**Supplementary Information:**

The online version contains supplementary material available at 10.1186/s12885-024-12861-y.

## Background

Gastric cancer (GC) is a prevalent disease worldwide, ranking fifth in incidence and fourth in cancer-related mortality [1]. Advanced GC patients possess a median survival time of 12 to 15 months [2]. The past few years have witnessed a burgeoning interest in immunotherapy and targeted medicine due to the limitations of traditional treatment regimens regarding efficacy and indications. However, immunotherapy’s response rates in advanced GC have not been satisfactory, ranging from 10–26% [3–5]. Recent research has discovered that the intricate nature and heterogeneity of the tumor microenvironment (TME) account for varying immunotherapy responses. Researchers identified that the tumor’s hypoxic environment causes a high glycolytic metabolism that might produce excessive lactate. This excessive lactate environment boosts PD-1 expression and Treg cell suppressive activity, partly explaining why PD-1 blocking therapy is ineffective [6]. Importantly, exploring the intricate TME can open the door to novel therapeutic approaches and enable accurate therapeutic outcome prediction.

Breakthroughs in single-cell and spatial transcriptome technologies over the past four years have made it possible to explore the heterogeneity of the TME at high resolution. In triple-negative breast cancer (TNBC), quiescent cancer cells create an ecological niche containing immunosuppressive fibroblasts, dysfunctional dendritic cells, and highly depleted T cells [7]. Eliminating these cells is anticipated to counter immunotherapy resistance in TNBC and prevent cancer recurrence. Consequently, there is an urgent need for researchers to explore the tumor microenvironment of gastric cancer using single-cell analysis, spatial transcriptome analysis, and multiplex immunohistochemistry technology. Indeed, a deeper understanding of TME heterogeneity could contribute to discovering novel therapeutic strategies to control the GC progression by exploiting TME regulatory weaknesses and reprogramming TME components.

Cancer-associated fibroblast (CAF) is a prominent stromal cell subpopulation implicated in cancer progression and metastasis. CAFs can support cancer cells by remodeling the extracellular matrix (ECM), promoting angiogenesis, and maintaining a chronic inflammatory state. Although various studies have examined CAF-related signaling pathways by bulk RNA sequencing, the effector function and heterogeneity of the CAFs in GC remain unknown [8]. A POSTN^high^ extracellular matrix CAFs (eCAFs) subpopulation has been identified by researchers using single-cell analysis. This eCAF subpopulation expressed genes involved in ECM remodeling at high levels, which directly impacted GC progression [9]. A CAF subpopulation with marked CTHRC1 expression in GC was discovered, which was overexpressed in tumor tissues compared to normal one and mediated tumor progression [10]. Previous studies on fibroblasts in GC had limited cell counts and could not provide a comprehensive view of the entire fibroblast landscape. Additionally, the TME of GC exhibits intricate crosstalk among various cell clusters, creating a distinct organic ecosystem. As a result, the communication between fibroblasts and other cell types within GC TME remains poorly understood and requires further investigation.

Herein, we observed the intricate nature and heterogeneity of the GC TME and the metabolic properties of major cell types. Furthermore, two cell subtypes, LOX^+^ Fibroblasts and M2 Macrophages, were enriched in tumor tissue and associated with the survival of GC patients. The two cell subpopulations had a strong correlation and were both present in a hypoxic microenvironment. Of note, LOX^+^ Fibroblasts may act as a trigger to stimulate monocyte differentiation into M2 Macrophages via IL6-IL6R. Taken together, our findings reveal the complex cross-talk between fibroblast and macrophage subpopulations, which provides novel insights for manipulating the TME.

## Materials and methods

### Collection and pre-processing of single-cell sequencing

The raw expression matrix of scRNA-seq was acquired from Gene Expression Omnibus (GEO, https://www.ncbi.nlm.nih.gov/geo/ ) dataset GSE183904 [11], which includes 10 paracancerous tissues and 26 tumor ones. As the GEO database is a freely accessible resource, ethical approval was not required.The quality control (QC) process was performed using the Seurat R package, which included all samples. Cells were excluded if they met any of the subsequent criteria: (A) less than 500 genes detected; (B) greater than 6000 genes detected; and (C) mitochondrial gene expression percentages exceeded 20%. Subsequently, the study focused on the single cells that passed the filtering criteria.

### Bulk RNA expression matrix acquisition

The publicly available normalized gene expression data for gastric cancer was collected from The Cancer Genome Atlas (TCGA, https://www.cancer.gov/ccg/research/genome-sequencing/tcga ) database. 375 GC patients were eventually allowed to the next analysis after the samples with no follow-up were eliminated. Additionally, RNA-seq expression matrix of GC patients based on Affymetrix microarrays, including GSE15459 [12], GSE26253 [13], GSE66229 [14], GSE84426, and GSE84437, were collected from the GEO database. Gene-expression values were log2 transformed and normalized by quantile normalization if the matrix was not previously normalized.

### Single-cell analysis

After the QC process described above, 150,913 single cells were enrolled in dimension reduction and clustering analysis. The single-cell sequencing matrix was normalized using the “LogNormalize” method and a scale factor of 10,000 based on Seurat R package. The Seurat’s CellCycleScoring function was used to determine each cell’s cell-cycle score. To eliminate the effects of cell-cycle-related noise and the mitochondrial content percentage, Seurat’s ScaleData function was used to perform regression. Next, the harmony R package was used to eliminate the batch effect. The top 50 principal components and the top 3000 variable genes were selected for cell clustering and the uniform manifold approximation and projection (UMAP) visualization. Marker genes for each cell cluster were identified through the FindAllMarkers function, and the single cells were clustered at two phases of the analysis. During the first phase, all cells were categorized and classified into the following cell types: epithelial cells (EPCAM, CD1H), T/NK cells (CD3D, CD3E), plasma B cells (CD79A, MZB1), endothelial cells (VWF, PLVAP), monocyte/macrophages (CD14, CD163), fibroblasts (DCN, COL1A1), B cells (CD19A, CD79A) and Dendritic cell (CD1C). A cell cluster is classified as a doublet cluster if it exhibits a significant expression of marker genes from different cell types. In the second analysis phase, specific cell-type clusters were extracted and subjected to re-clustering. Clusters with cell counts exceeding 200 will be annotated based on previously reported specific marker genes or cellular signature markers associated with those cell types. Monocle 2 R package [15] is a robust tool designed for analyzing the evolutionary trajectory of cells. In our study, we performed pseudo-time analysis to reconstruct the monocyte/macrophage evolution trajectory in gastric cancer.

### Cell-type infiltration based on single-cell and bulk RNA sequencing

To determine the infiltration of the defined cell types, we utilized single-sample gene set enrichment analysis (ssGSEA), a reliable method for estimating cell-type infiltration. Firstly, we employed the FindAllMarkers function to identify marker genes for each cell type. Secondly, we selected the top 10 marker genes for each cell type to create a specific gene set for that particular cell type. For example, the gene set for mast cells included genes such as PSAB1, TPSB2, CPA3, TPSD1, GATA2, LTC4S, MS4A2, HPGDS, KIT and CLU. Subsequently, we employed the “GSVA” and “GSEABase” R packages to calculate the infiltration proportion of each predefined cell type in the TCGA and GEO databases. The ssGSEA score for each cell type was normalized.

A survival analysis was conducted using the survival R package. The survfit function was used to model the Kaplan-Meier survival curve. The “surv cutpoint” function of the survminer R package was applied to determine the best-cutoff point. The optimal cutoff point for gene expression or cell type infiltration fraction was used to classify patients into two groups. The Kaplan-Meier survival curves were compared based on the two-sided log-rank test. Furthermore, we conducted Spearman’s correlation analysis to calculate the relationships between the proportions of cell-type infiltration, considering a significant correlation at a false discovery rate (FDR) of 0.05. To visualize the correlation between cell infiltrations among different cell types in gastric cancer, we utilized the ggpubr R package.

### Functional enrichment analysis

Differentially expressed genes (DEGs) of single-cell sequencing were found by the FindMarkers function. The DEGs were subjected to Gene Ontology (GO) pathway enrichment and Kyoto Encyclopedia of Genes and Genomes (KEGG) analysis using the clusterProfiler R package [16]. To assess whether a predefined gene set exhibited significant differences between two cell clusters, Gene Set Enrichment Analysis (GSEA) [17] was performed. The Molecular Signatures Database (MSigDB) was employed to retrieve the KEGG gene sets. Gene Set Variation Analysis (GSVA) was also utilized to determine the expression scores of 50 hallmark pathways among the cell clusters. The pathway enrichment fraction of single cells was calculated using the Seurat package’s addModuleScore function, with the gene sets derived from the 50 hallmark pathways obtained from GSEA | MSigDB | Human MSigDB Collections (https://www.gsea-msigdb.org/gsea/msigdb/human/genesets.jsp?collection=H).

### Transcription factor regulon analysis

The SCENIC R package [18]was utilized to create gene regulatory networks that could identify transcription factors and cell states. To achieve this, co-expression modules between transcription factors and candidate target genes were determined using the GENIE3 method. A regulon was identified for each transcription factor and its target genes. The gene signature was enhanced using the cisTarget human motif database, and targets in this signature were pruned based on the default set of cis-regulatory cues. Then, using the AUCell algorithm, each regulon’s activity in each cell was assessed. The ComplexHeatmap and Heatmap R packages were employed to visualize the results.

### Immunohistochemistry and immunofluorescence staining

Nine pairs of paraffin-embedded GC tissue and adjacent samples were obtained from Tianjin’s first central hospital. Patients included in this study did not receive preoperative radiotherapy or chemotherapy. More crucially, all patients have approved the use of the surgical material for academic research and publications. All paraffin-embedded specimens were gathered in line with the ethical standards of The Institutional Review Committee and the Medical Ethics Committee of Tianjin first central hospital. Written informed consents have been obtained from all subjects. Paraffin-embedded tissues were examined by IHC staining according to standard immunoperoxidase staining procedures. The slides were incubated with anti-LOX (1:100; DF13251, Affinity, Rabbit) and anti-CD206 (1:1000; GB113497, Servicebio, Rabbit), following the manufacturer’s protocol. The slide images were independently examined and quantified by two pathologists. The IHC intensity score is 0 (negative), 1 (weak brown), 2 (medium brown), or 3 (strong brown). The staining content was categorized into five levels: 0 (≤ 10%), 1 (11-25%), 2 (26-50%), 3 (51-75%), or 4 (> 75%). The staining value was determined by multiplying the intensity scores and extent scores. Based on this value, the staining was further classified into three categories: weakly positive (1–3), positive (4–6), and strongly positive (7–12). In the present study, double-labeled immunofluorescence staining was performed using TSA (Tyramide signal amplification) Technology. Briefly, microwave processing was used for antigen retrieval, followed by sealing the tissue with 3% BSA. We then added the first primary antibody and incubated overnight. After adding the corresponding HRP-labeled secondary antibody, TSA was added dropwise and incubated for 10 min at room temperature away from light. Then, microwave processing was performed to remove the primary and secondary antibodies. The above steps were repeated for the next antibody. After that, a DAPI stain was applied to re-stain the cell nuclei. Finally, the samples were observed and photographed under fluorescent microscopy.

### Quantitatively characterizing cell-cell communications

In order to investigate the interaction between LOX^+^ Fibroblast and monocyte, we used NicheNet [19] to calculate the potential ligand-receptor pairs among cell types in GC TME. Ligands or receptors were assumed to be genes expressed in clusters with more than 10% of those cells. LOX^+^ Fibroblast and monocyte from normal tissues were regarded as reference sender cells and receiver cells, respectively. Ligand regulatory activity was plotted using the Nichenet output ligand activity target heatmap. Scores for activities varied from 0 to 1.

### Statistical analysis

R (4.10) and GraphPad Software (version 8.0) were employed for all statistical analyses. Statistical significance was defined as P-value < 0.05. The Wilcoxon test was performed for comparisons between the two groups. To compare three or more groups, one-way ANOVA was used.

## Results

### Cellular constitution of human normal gastric mucosa and cancer tissues

**Fig. 1:**
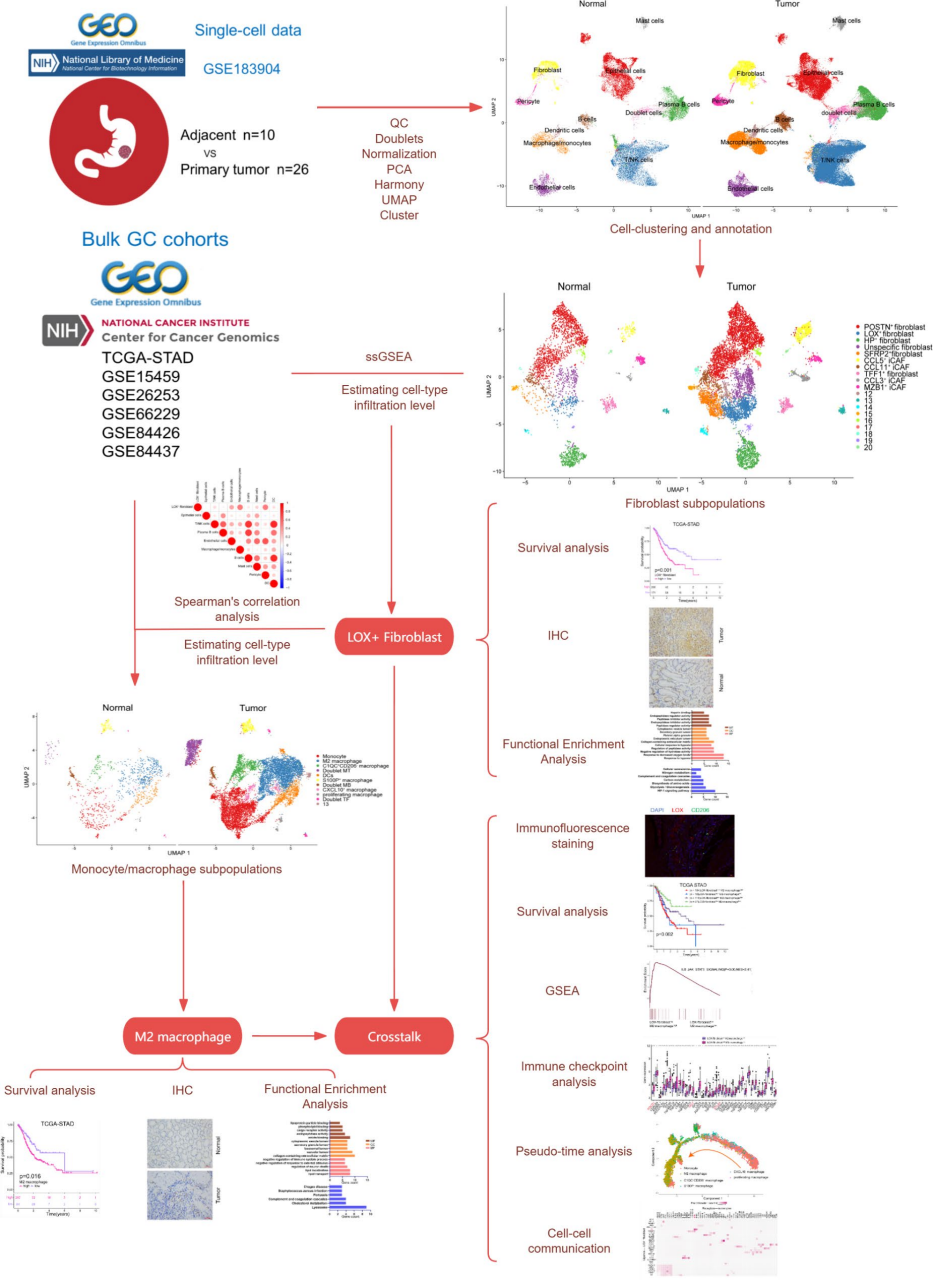
The workflow of the present study

**Fig. 2:**
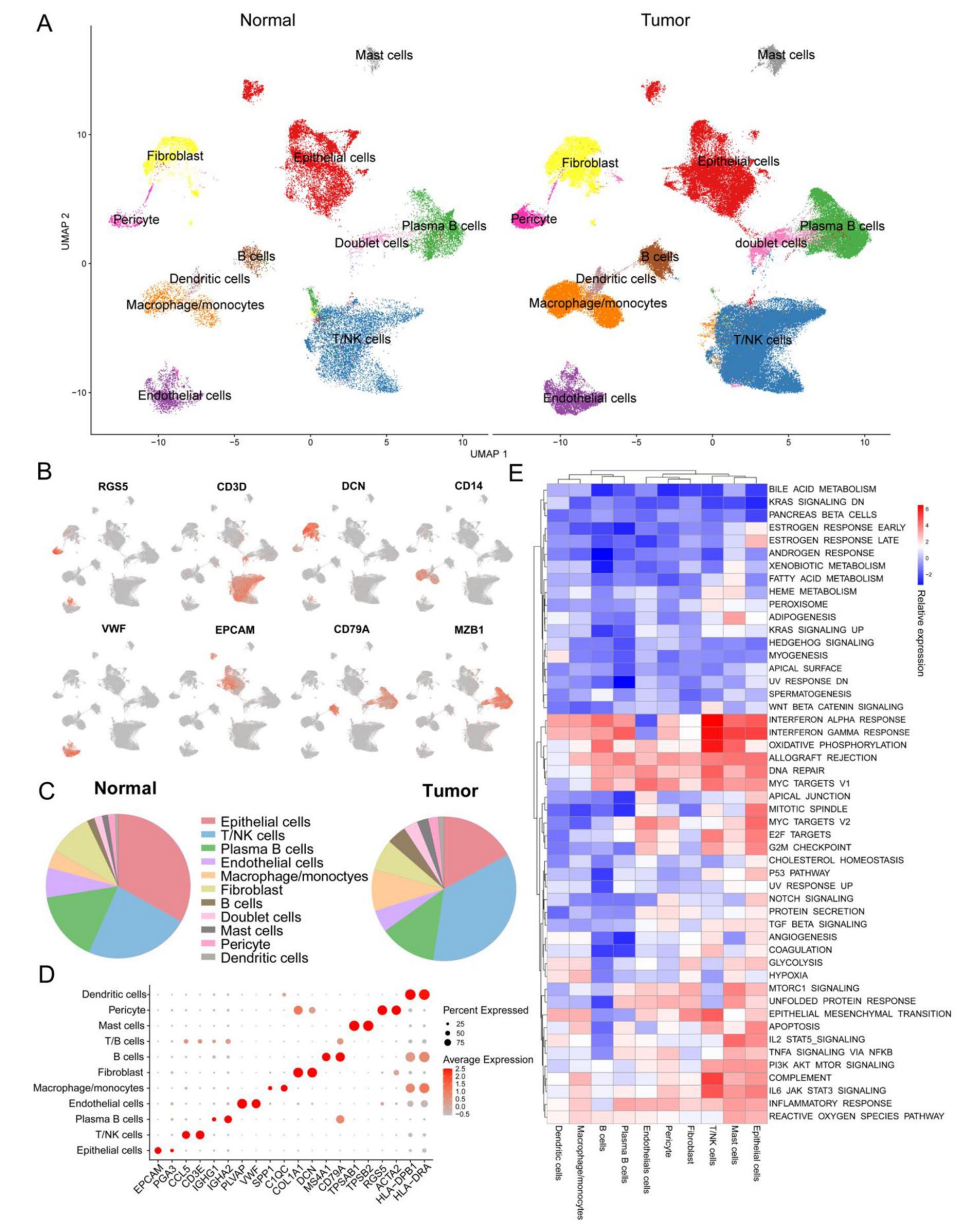
Cellular constitution of human normal gastric mucosa and cancer tissues. **(A)** UMAP plots of 150,913 single cells of 36 GC patients, displaying 10 cell type. **(B)** The expression levels of selected known marker genes in 150,913 cells from both normal and malignant tissue are depicted in UMAP plots. **(C)** Pie chart of the distribution of all identified cell types: the proportion of each cell in normal and tumor tissue. **(D)** Dot plots of the top two marker genes of 10 major cell types identified in this profile. **(E)** Dot plots of 50 hallmarks pathway activities score in the cell types between tumor and adjacent normal tissues

As shown in Fig. 1, the flow chart presented the detailed procedures of this study. First, we conducted a single-cell analysis of 10 paracancerous tissues and 26 gastric cancer samples to obtain a scRNA-seq atlas of normal gastric mucosa and cancer samples. After applying the quality control procedures, we collected 150,913 single cells that met our quality criteria. The detected gene numbers, the depth of sequencing and the gene ratio of mitochondrial for each specimen were displayed in Figure S1. Of these cells, 31,150 were derived from normal gastric mucosa, while 119,763 cells came from tumor tissue (Fig. 2A). All cells were organized into 11 major cell clusters. These clusters were then annotated with classic biomarkers, including epithelial cells (*n* = 30,841) identified by the expression of EPCAM and CDH1, T/NK cells (*n* = 49,532) which expressed the CD3D and CD3E, B cells (*n* = 5735) marked by CD19 and CD79A, plasma B cells(*n* = 20,181) identified by CD79A and MZB1 expression, monocyte/macrophage cells (*n* = 12,336) which were positive for CD14 and CD163 expression, mast cells (*n* = 3429) defined by their classical markers TPSB2 and MS4A2, endothelial cells (*n* = 8046) marked by PECAM1 and VWF, fibroblast (*n* = 11,470) marked by COL1A1 and DCN, pericyte cells (*n* = 3188) marked by RGS5 and ACTA2, and Dendritic cell(DC, *n* = 1772 ) marked by CD1C. In addition to the 10 cell types above, we identified a doublet cell cluster, which highly expressed both T cell and B cell marker genes (Fig. 2B). Figure 2D depicts the top two markers of each cell type. Although all cell types were present in both normal mucosa and malignancies, their proportions were not equal (Fig. 2C). For instance, the proportion of T cells derived from tumor tissue was greater compared to normal tissue.

To investigate changes in functional pathways during the TME formation, we employed GSVA to identify alterations in the scores of 50 Hallmark pathways across various cell types. This analysis was performed to compare the adjacent normal and tumor tissues and elucidate the differential pathway activity in each cell type within the TME (Fig. 2E). Tumor-infiltrating immune and cancer cells exhibited greater enrichment of Interferon response than cells from normal mucosa. In addition to T cells and macrophages, which were enriched in immune-related pathways including IL6-JAK-STAT3 signaling and inflammatory responses, fibroblasts were also involved in the immune response to malignancies. Interestingly, hypoxia and glycolysis pathways were highly enriched in cancer cells, fibroblasts, and monocyte/macrophage cells from tumors than in normal tissues. This pattern might reflect specific hypoxic regions inside the tumor mass, which affects the metabolic pathways of the infiltrated tumor epithelial cells, fibroblasts, and monocyte/macrophages. In conclusion, the functional pathways of different cell types were reshaped in the GC TME.

### Tumor-infiltrating LOX+ fibroblasts was associated with tumor progression

**Fig. 3:**
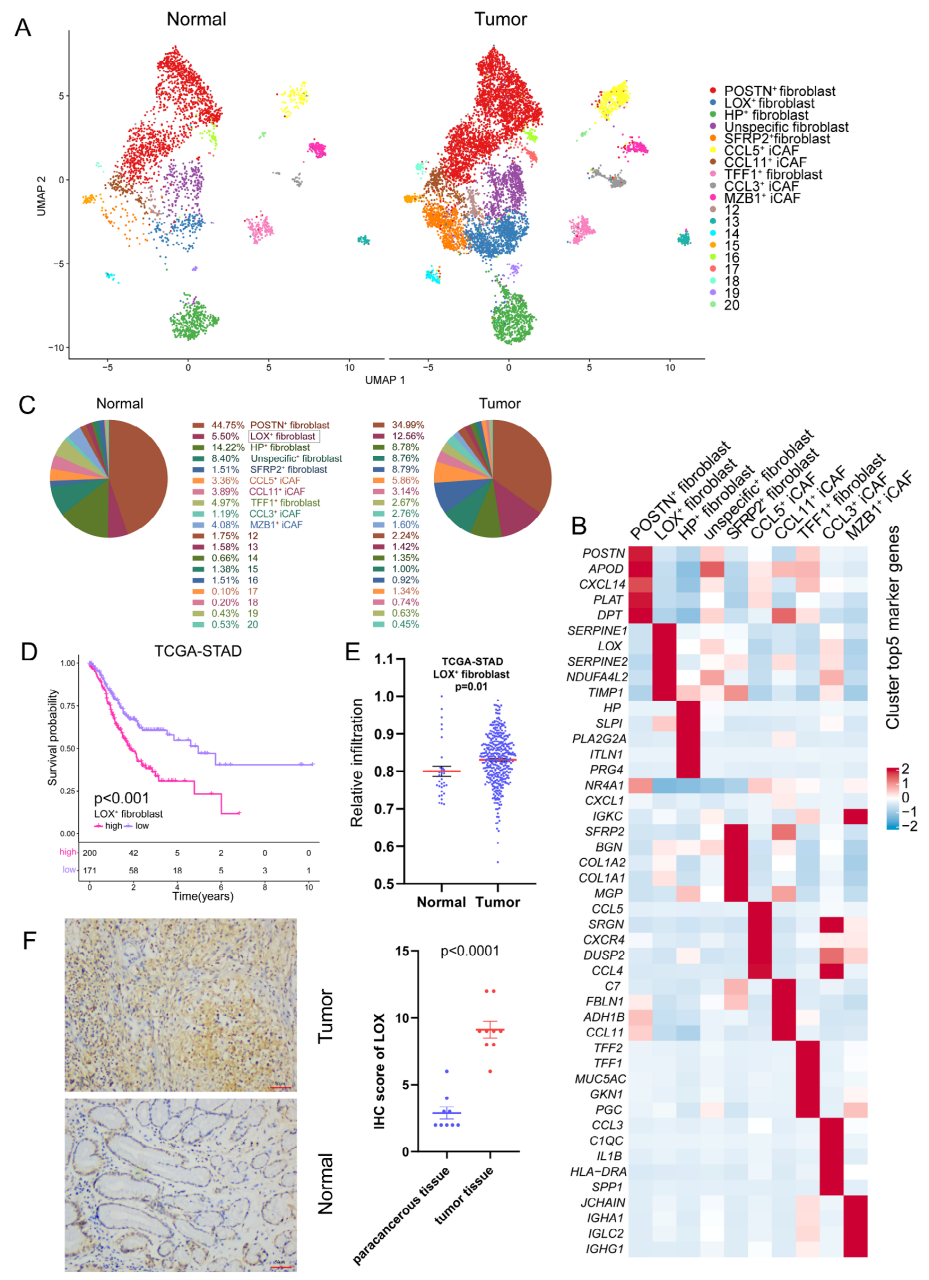
Tumor-infiltrating LOX^+^fibroblasts was associated with tumor progression. **(A)** UMAP plot of the subpopulations of fibroblasts colored by cluster. **(B)** Heatmap of the top five marker genes in each fibroblast subpopulations. **(C)** The proportion of fibroblast subpopulations in normal and tumor tissue. **(D)** The Kaplan–Meier overall survival curves of TCGA-STAD patients classified by LOX^+^fibroblasts infiltration. **(E)** Comparison of relative infiltration proportion of LOX^+^fibroblasts between normal and tumor tissue in TCGA-STAD matrix. **(F)** The expression value of LOX in GC tissue and adjacent normal specimens determined by IHC analysis

Recently, several studies have focused on developing prognostic models for GC based on fibroblast signature genes or calculating the degree of fibroblast infiltration using deconvolution algorithms [20, 21]. These investigations, however, have overlooked the heterogeneity of tumors. Fibroblasts are not a homogeneous cell population; they consist of multiple subpopulations distributed throughout the tumor and possessing distinct characteristics. It was recently proposed that fibroblasts could be broadly classified as immunomodulatory CAF (iCAFs), eCAF, and Myofibroblastic CAFs(myCAFs) [22, 23]. Alternatively, single-cell sequencing can define subpopulations based on markers specific to each fibroblast subpopulation [9, 10]. We investigated ~ 10,000 fibroblasts using single-cell data from 36 samples. We employed their specific marker genes or cellular signature markers reported before to re-cluster all fibroblasts into 10 subpopulations. Clusters with cell counts under 200 were not annotated as described in the method. If the cluster highly expressed genes for inflammatory mediators, we classified it as an iCAFs. Taken together, we identified 10 major fibroblast subtypes in normal mucosa and tumor sample (Fig. 3A and B), including POSTN^+^ fibroblast (*n* = 5304), LOX^+^ Fibroblast (*n* = 1226), HP^+^ fibroblast (*n* = 1172), SFRP2^+^ fibroblast (*n* = 787), unspecific fibroblast (compared to other clusters, no specific marker genes, *n* = 852), CCL5^+^ iCAF (*n* = 596), CCL11^+^ iCAF (*n* = 383), TFF1^+^ iCAF (*n* = 376), CCL3^+^ iCAF (*n* = 269) and MZB1^+^ iCAF(*n* = 259). Among them, LOX^+^ Fibroblasts exhibited higher LOX gene expression than other fibroblast subpopulations. In addition, previous single-cell analysis studies have identified the presence of LOX^+^ Fibroblasts [24, 25]. therefore, we defined them as LOX^+^ Fibroblasts. We found that LOX^+^ Fibroblasts were significantly enriched in tumor tissue compared to normal mucosa by evaluating the composition of each fibroblast subtype (Fig. 3C).

To further validate the changes in LOX^+^ Fibroblast, we computed the infiltration proportions of cell types in TCGA STAD (Stomach adenocarcinoma) and GSE66229-STAD cohorts described in the methods due to the single-cell data’s sample size restrictions and variations in the number of cells detected in a single-cell sample. Consistent with our previous result, the abundance of LOX^+^ Fibroblast in tumor tissue was substantially higher than in nearby normal one (Fig. 3E, Figure S2A). Immunohistochemical results discovered that LOX (representing the LOX^+^ Fibroblasts) was significantly overexpressed in GC tissues compared to the corresponding non-cancerous normal tissues (Fig. 3F). Interestingly, GC patients with higher relative infiltration of LOX^+^ Fibroblast had short OS in both TCGA and GEO cohorts (Fig. 3D, Figure S2B).

### Hypoxia-related pathways were enriched in LOX+ fibroblasts

**Fig. 4:**
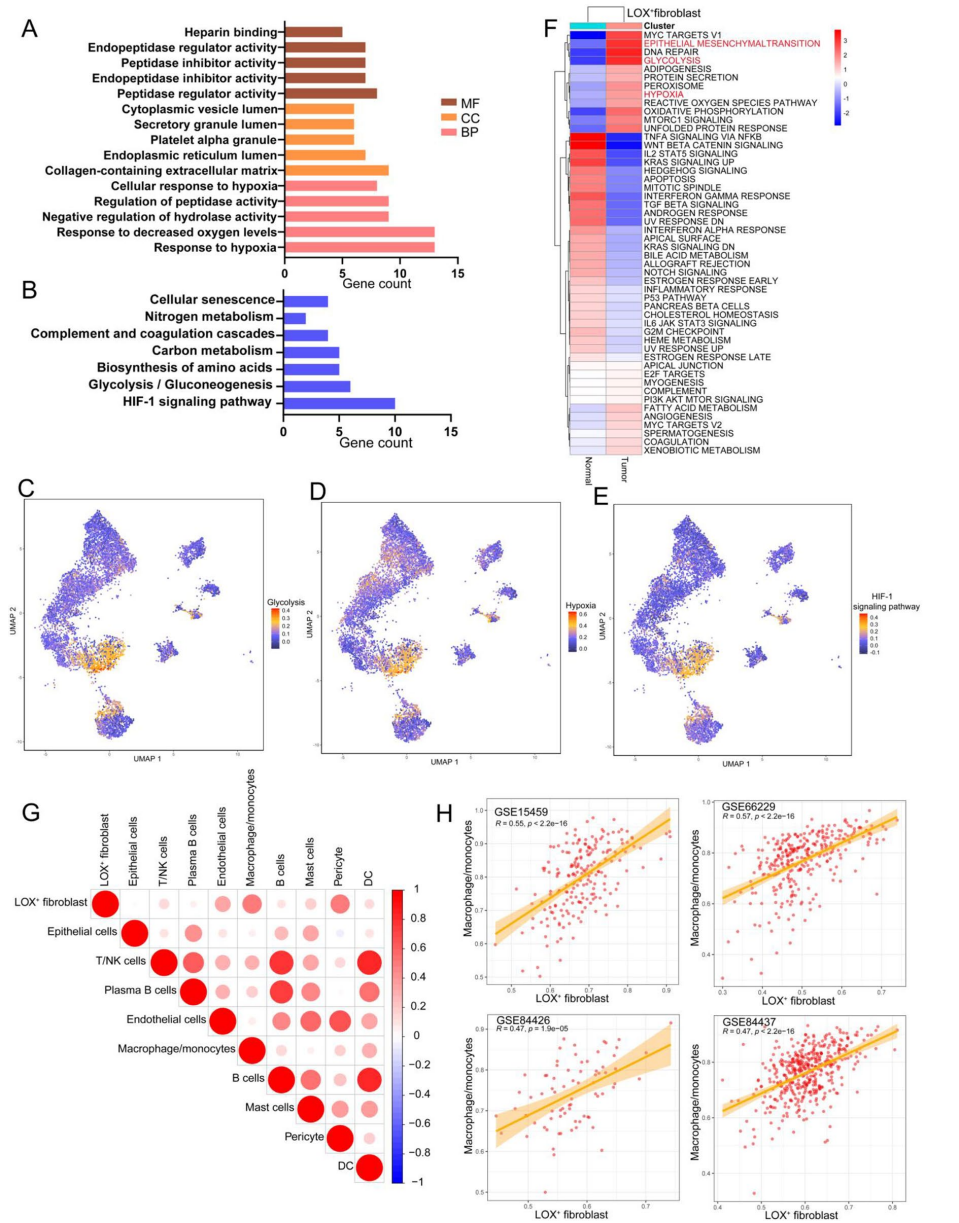
LOX^+^ Fibroblast showed enrichment of hypoxia-related pathways. **(A)** Gene Ontology (GO) and KEGG **(B)** analysis of the specific genes of LOX^+^ fibroblasts. **(C)** The enrichment score for the glycolysis **(C)**, hypoxia **(D)** and HIF-1 A signaling pathways **(E)** in fibroblast subpopulations, which was calculated by Seurat’s addmodulescore function. **(F)** Differences in 50 hallmark pathway activities scored with GSVA. A linear model’s t values are displayed. **(G)** Plot showing the spearman correlations within the infiltration patterns of LOX^+^ Fibroblasts and other nine major cell types in TCGA STAD cohorts (positive correlations in red, negative correlations in blue). **(H)** Scatter plots unveiled the correlation between the infiltration of LOX^+^ Fibroblasts and other major cell types across 4 independent GC datasets, including GSE66229, GSE15459, GSE84426 and GSE84437. The error band indicates 95% confidence interval

To examine the effector function of LOX^+^ Fibroblasts, we analyzed the DEGs between LOX^+^ Fibroblasts and other fibroblast subpopulations using the FindAllMarkers function and performed GO and KEGG analysis. GO analysis showed that the response to hypoxia and response to decreased oxygen levels were associated with LOX^+^ Fibroblasts (Fig. 4A). Furthermore, LOX^+^ Fibroblasts showed enrichment of the HIF-1 signaling pathway and Glycolysis/Gluconeogenesis (Fig. 4B). It is well-established that hypoxic regions promote abnormal angiogenesis, adhesion proliferation, and inflammation. Additionally, hypoxia ruins the environment necessary for immune cells to survive and disrupts important regulatory processes, making cancers immune-resistant to immunotherapy [26]. Using Seurat’s addmodulescore function, we determined each fibroblast’s enrichment score for the hypoxia-related pathways, including the glycolysis, HIF-1 A, and hypoxia pathways. Consistent with the GO and KEGG results, LOX^+^ Fibroblasts exhibited higher hypoxia-related pathway scores than other fibroblast subtypes (Fig. 4C, D and E). Importantly, our single-cell sequencing data included both normal and cancer tissues, allowing us to investigate how LOX^+^ Fibroblasts change during TME formation. Using the 50 hallmark gene sets, we discovered that hypoxia and the glycolysis regulatory network were significantly enriched in LOX^+^ Fibroblasts in tumors compared to normal tissues. In tumor-derived LOX^+^ Fibroblasts, the epithelial-mesenchymal transition was also upregulated (Fig. 4F).

The aforementioned analysis revealed that low-oxygen regions within the tumor could impact the metabolic pathways of infiltrating tumor epithelial cells, fibroblasts, and monocyte/macrophages. By studying fibroblasts at the subtype level, we confirmed that the fibroblasts in these hypoxic regions were LOX^+^ Fibroblasts. To investigate the relationship between LOX^+^ Fibroblasts and other cell populations in the GC microenvironment, we calculated Spearman correlations between their infiltration patterns and those of the other nine major cell types in TCGA STAD cohorts. We discovered a significant positive correlation between LOX^+^ Fibroblasts and monocyte/macrophage (*R* = 5.1, *p* < 0.05) (Fig. 4G). This relationship was also confirmed in the other 4 independent GEO STAD cohorts (Fig. 4H). As a result, we hypothesize that LOX^+^ Fibroblasts and monocyte/macrophages interact in hypoxic regions, possibly influencing tumor progression.

### Tumor-infiltrating M2 macrophage was associated with tumor progression

**Fig. 5:**
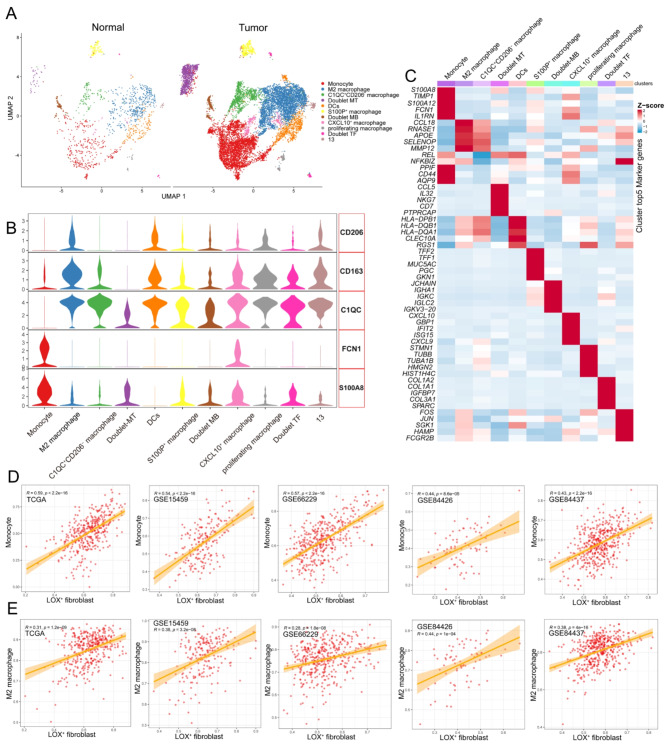
Tumor-infiltrating M2 Macrophage was associated with tumor progression. **(A)** UMAP plot displaying the subpopulations of macrophage/monocytes. **(B)** violin plots depicting the expression value of specific genes. Colors as in A. **(C)** Heatmap of the top five marker genes in each macrophage/monocyte subpopulations. **(D)** Scatter plots illustrate the relationship between the infiltration of LOX^+^ Fibroblasts and monocytes **(D)** or M2 Macrophages **(E)** across 5 independent GC datasets, including TCGA-STAD, GSE66229, GSE15459, GSE84426 and GSE84437

**Fig. 6:**
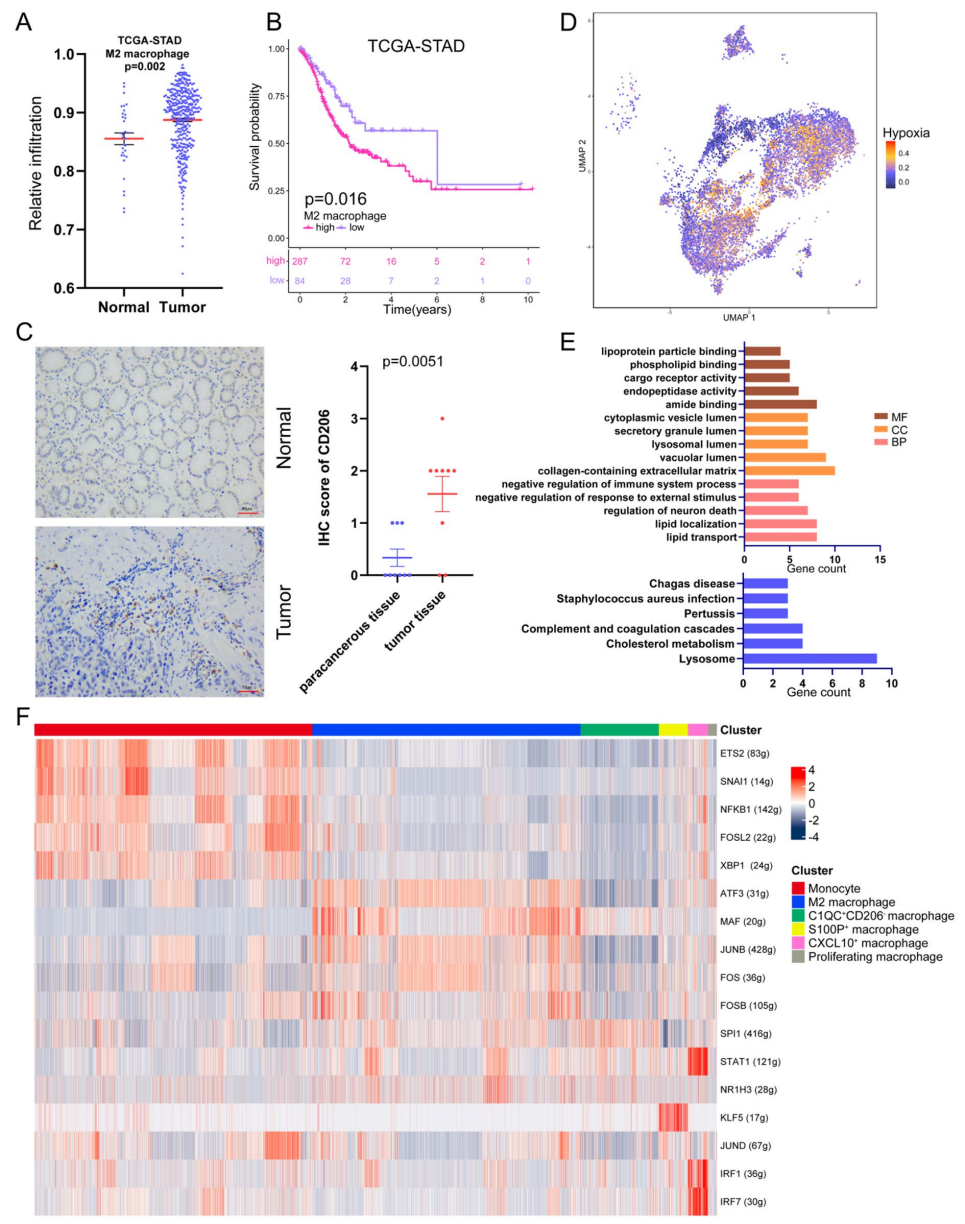
**(A)** Comparison of relative infiltration proportion of M2 Macrophages between normal and tumor tissue in TCGA-STAD cohort. **(B)** The Kaplan–Meier overall survival curves of TCGA-STAD patients classified by M2 Macrophages infiltration. **(C)** The expression value of CD206 in GC tissue and adjacent normal specimens determined by IHC analysis. **(D)** The enrichment score for the hypoxia pathways in monocyte and macrophage subpopulations. **(E)** GO (upper) and KEGG (bottom) analysis of the specific genes of M2 Macrophages. **(F)** Heatmap shows the AUC scores of the expression of the SCENIC-identified transcription factors

We then clustered all monocyte/macrophage into 9 cell clusters and observed 3 doublet cell clusters, including macrophage/T cell doublet (MT, *n* = 987), macrophage/B cell (MB, *n* = 310), and T cell/fibroblast (TF, *n* = 128) doublet clusters. Additionally, we discovered a DC subtype (*n* = 883) with prominent HLA-DPB1 and HLA-DQB1 expressions. In the next study, we did not consider the doublet clusters and DC subtype mentioned earlier, as our focus was on monocytes/macrophages. Our analysis identified six subtypes, including Monocyte (*n* = 4034),M2 macrophage (*n* = 3891), CQ1C^+^MRC1^−^ macrophage (*n* = 1136), S100P^+^ macrophage (*n* = 421), CXCL10^+^ macrophage (*n* = 291), and proliferating macrophage (*n* = 129) Fig. 5A). Monocytes exhibited high expression of FCN1 and S100A8, typical monocyte biomarkers. The M2 macrophages were characterized by C1QC, CD163, and CD206(MRC1) expression, markers of M2 TAM [27, 28]. CQ1C^+^MRC1^−^ macrophages were positive for C1QC and CD163 but not for CD206. S100P was highly expressed in S100P^+^ macrophage, while CXCL10 was upregulated in CXCL10^+^ macrophage (Fig. 5B and C). Furthermore, we observed a proliferating macrophage subtype marked by TOP2A and MKI67 expression. To further investigate the relationship between LOX^+^ Fibroblasts and monocyte/macrophage, we utilized ssGSEA to calculate the infiltration of 6 subtypes above identified by scRNA-seq and further calculated the Spearman correlations between LOX^+^ Fibroblasts and other 6 macrophage subtypes in each cohort. The LOX^+^ Fibroblasts were most strongly correlated with either monocytes or M2 Macrophage (Fig. 5D and E). Therefore, we investigated the changes in infiltration levels of M2 Macrophages between adjacent tissues and tumor tissues. Tumor tissue had a considerably higher concentration of M2 Macrophages than nearby normal tissue (Fig. 6A). Immunohistochemical results discovered that CD206 (representing M2 Macrophages) was significantly overexpressed in GC tissues compared to the corresponding non-cancerous normal tissues (Fig. 6C). The Kaplan-Meier survival curve displayed GC patients with higher M2 Macrophage infiltration exhibited short OS (Fig. 6B).

### The progression of gastric cancer is accelerated by LOX+ fibroblasts and M2 macrophages interactions

Our results suggested a strong interaction existed between LOX^+^ Fibroblasts and monocytes/macrophages in the hypoxic TME. Hence, we calculated the hypoxia pathway enrichment score of each macrophage subtype using the addmodulescore function in Seurat. As expected, the hypoxia pathway was significantly enriched in monocyte and M2 Macrophages (Fig. 6D), which confirmed our speculation. We hypothesized the presence of a highly coordinated network of LOX^+^ Fibroblasts and M2 Macrophages in the hypoxic region, which works synergistically to exacerbate the GC microenvironment. Furthermore, pseudotime analysis predicted that M2 Macrophages probably originated from monocytes. We then used the FindAllMarkers function to evaluate the DEGs and conducted GO and KEGG analyses to investigate the role of M2 Macrophages in GC because they are M2 TAMs. In addition to the M2 marker genes mentioned above, we found significant expression of CCL18 (C-C Chemokine Ligand 18) and APOE (Apolipoprotein E) in M2 Macrophages. CCL18 is a chemokine secreted by macrophages and dendritic cells, which can induce T regulatory cell differentiation and recruitment into the TME and subsequently induces an M2-like macrophage phenotype [29, 30]. ApoE exosomes derived from tumor-infiltrating M2 macrophages can activate PI3K-AKT signaling and promote cytoskeletal remodeling, increasing GC cell migration potential [31]. GO analysis revealed lipid-metabolism-related pathways, like lipid transport and lipid localization, were enriched in M2 Macrophages (Fig. 6E). Furthermore, M2 Macrophages were related to negative regulation of the immune system process and response to external stimulus. Moreover, M2 Macrophages were potentially regulated by Cholesterol metabolism. Macrophages with high lipid metabolism activity were identified in liver and breast cancers [32, 33], which enhanced the accumulation and polarization of M2 TAMs, induced immunosuppressive activity of TAMs, and promoted cancer progression. We used SCENIC analysis to discover the key regulator of M2 Macrophages (Fig. 6F). The results discovered that MAF, a typical marker associated with an M2-like pro-tumorigenic phenotype [34], was highly activated in M2 Macrophages. Furthermore, M2 Macrophages possessed the highest ATF3 expression, which was recently found to promote macrophage migration and reverse M1-polarized macrophages to the M2 phenotype [35]. Hence, ATF3 and MAF may be key regulators in converting monocytes to M2 Macrophages.

**Fig. 7:**
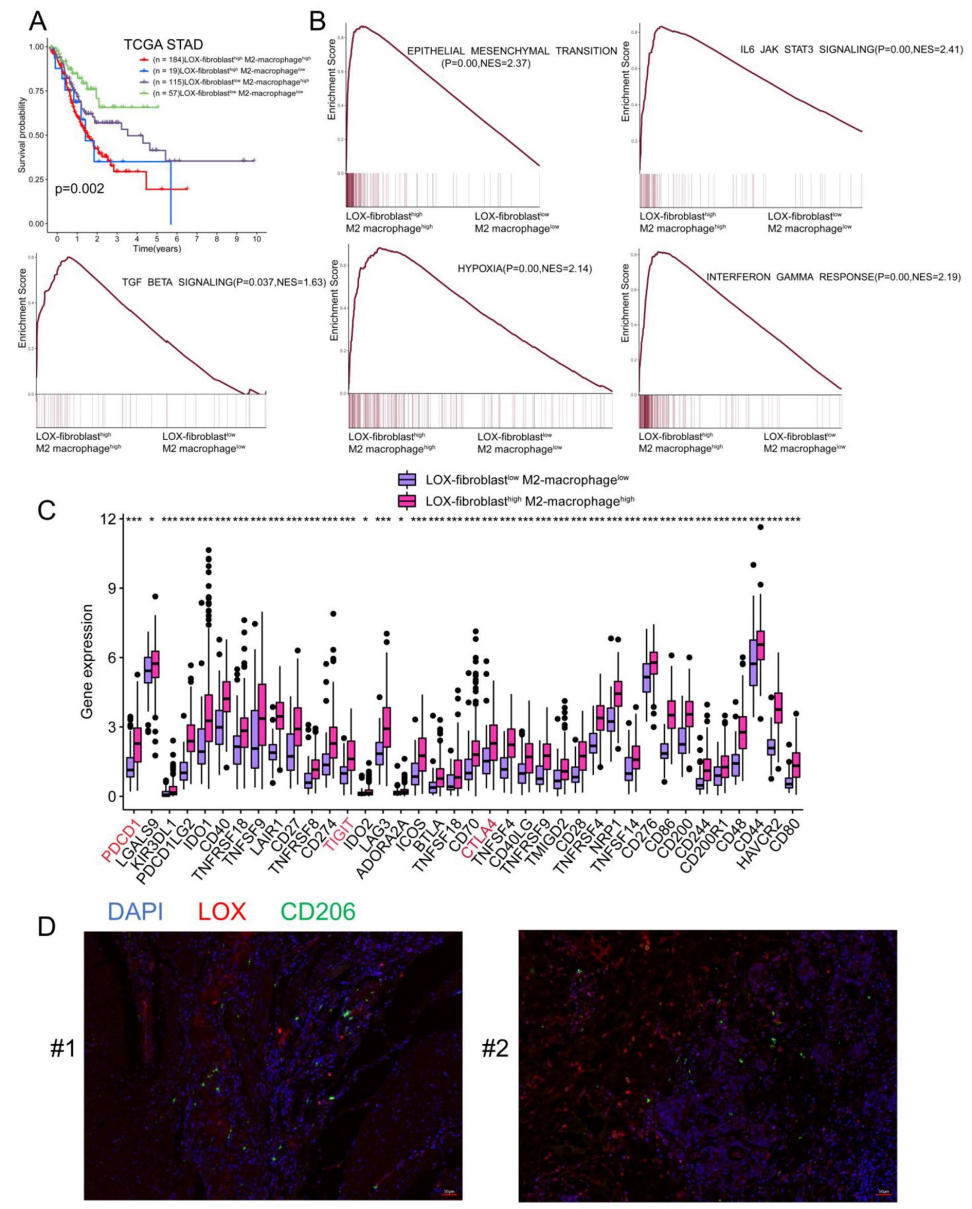
The progression of gastric cancer is accelerated by LOX^+^ Fibroblasts and M2 Macrophages interactions. **(A)** Overall survival analyses for four subgroups of TCGA-STAD patients classified by the infiltration of both LOX^+^ Fibroblasts and M2 Macrophages. **(B)** GSEA of hypoxia, TNF BETA signaling, hypoxia, interferon response, and IL6-JAK-STAT3 pathways between LOX-fibroblast^high^/M2-macrophage^high^ and LOX-fibroblast^low^/M2-macrophage^low^ groups. Ranking genes by fold change in expression between these two conditions. NES, normalized enrichment score. **(C)** Comparison of the immune checkpoint expression level between LOX-fibroblast^high^/ M2-macrophage^high^ and LOX-fibroblast^low^/ M2-macrophage^low^ groups. (D) Characterization of cell location with mIHC. A staining panel was developed to visualize DAPI (blue), CD206(green) and LOX (red) simultaneously on the same tissue slide. Experiment was performed in two independent patients

To explore the clinical significance of such a close correlation between LOX^+^ Fibroblasts and M2 Macrophages, we divided TCGA STAD patients into different groups based on the infiltration degree of LOX^+^ Fibroblasts and M2 Macrophages. The shortest OS was observed in patients with high LOX^+^ Fibroblasts and M2 Macrophages, suggesting these two cell types could work synergistically to precipitate tumor development (Fig. 7A). To investigate the interaction mechanism of the two cell types, we performed GSEA analysis between LOX-fibroblast ^high^ M2-macrophage^high^ group and LOX-fibroblast ^low^ M2-macrophage^low^ group. Genes upregulated in LOX-fibroblast ^high^ M2-macrophage^high^ group showed the enrichment of TGF BETA signaling and epithelial-mesenchymal transition. Additionally, these GC patients exhibited high enrichment in hypoxia gene sets. Furthermore, IL6-JAK-STAT3 signaling and Interferon GAMMA response were enriched in LOX-fibroblast ^high^ M2-macrophage^high^ group (Fig. 7B). Meanwhile, patients with high infiltration of both subpopulations had significantly higher immune checkpoint expression than those with low infiltration, implying that the two subpopulations interact to enhance the formation of an immunosuppressive environment (Fig. 7C). These findings imply that LOX^+^ Fibroblasts and M2 Macrophages could interact with one another in the GC TME. We further validated our *in silico* results in tissue samples from GC patients. We examined whether LOX^+^ Fibroblasts and M2 Macrophages were closely localized in GC tissues (Fig. 7D). The proximity of LOX-positive cells and CD206-positive cells was observed through immunofluorescent double-labeling, suggesting a possible interplay between these subpopulations.

### LOX+ fibroblast stimulate monocyte differentiation into M2 macrophages in a hypoxic environment

**Fig. 8:**
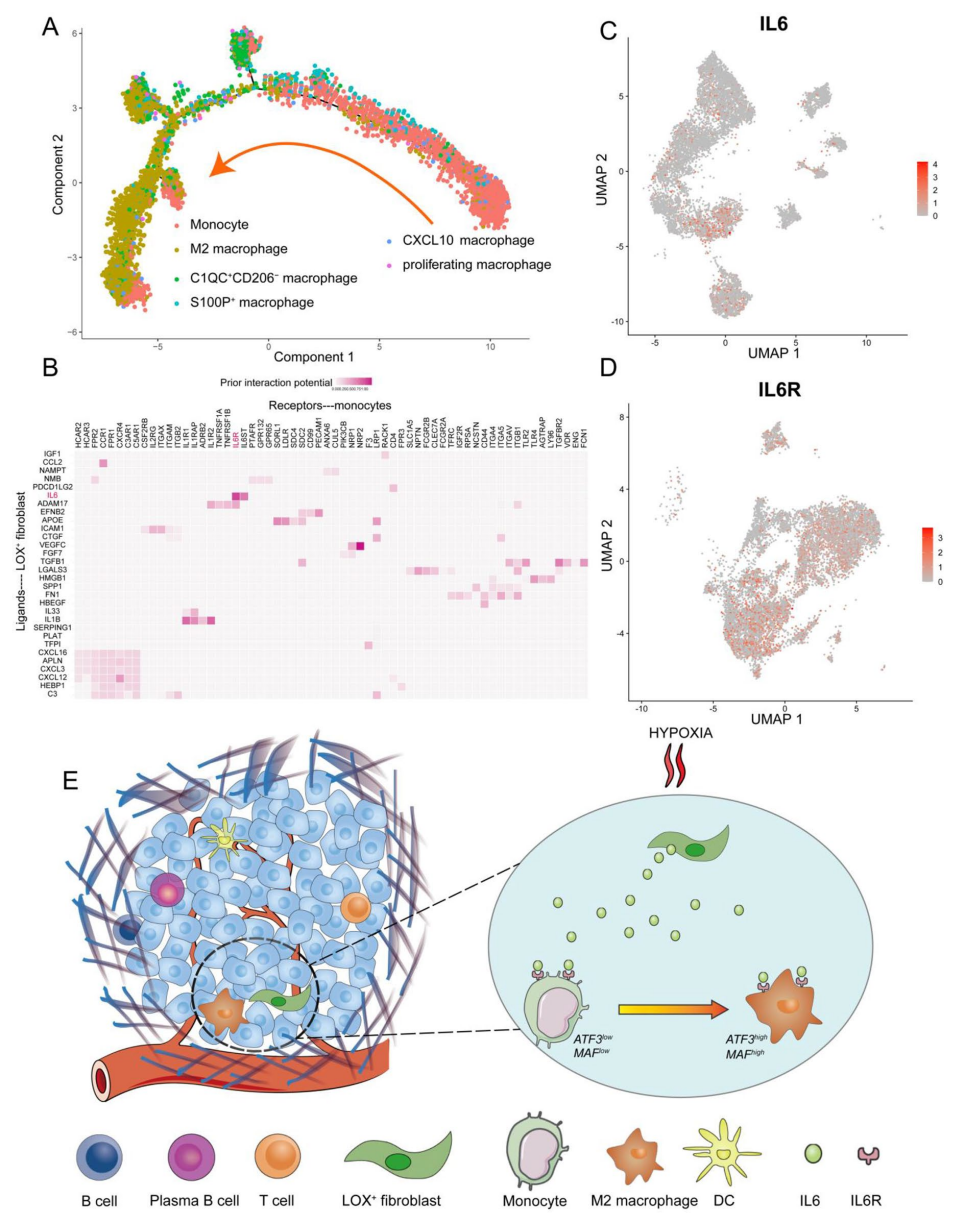
LOX^+^ Fibroblast stimulate monocyte differentiation into M2 Macrophages in a hypoxic environment. **(A)** Differentiation trajectory of monocytes and macrophage subpopulations, colored for cell subpopulations. The arrow indicates the direction of cell differentiation. **(B)** Top-ranked ligands and its corresponding receptors inferred to regulate M2 Macrophages by LOX^+^ Fibroblasts colored by ligand activity according to NicheNet. **(C)** UMAP plots exhibiting the IL6 expression value in fibroblast subtypes **(C)** and IL6R in myeloid cell subclusters **(D)**. **(E)** Working mechanism for the interaction between M2 Macrophages and LOX^+^ Fibroblasts in a hypoxia-immunosuppressive microenvironment

Studies have shown that macrophages are a subtype of myeloid cells differentiated from monocytes. According to pseudotime analysis, both M2 Macrophages and other macrophage subtypes originate from the differentiation of monocytes (Fig. 8A). To further identify the role played by LOX^+^ Fibroblasts in the differentiation of monocytes into M2 Macrophages, we examined the cell-cell communication mechanism of LOX^+^ Fibroblasts and monocyte. Using the NicheNet R package, we could calculate the ligand-receptor pairs among two cell types. LOX^+^ Fibroblast and monocyte from normal tissues were regarded as reference sender cells and receiver cells, respectively. There were many chemokine pairs between the two cell subpopulations, such as CCL2-CCR1, CXCL12-CXCR4, and others, suggesting the complexity of their crosstalk. Importantly, LOX^+^ Fibroblasts interacted with monocytes through IL6-IL6R (Fig. 8B), which exhibited the strongest ligand regulatory activity. Then, after examining the level of IL6 expression across all fibroblast subtypes, we discovered that LOX^+^ Fibroblasts expressed IL6 at a higher level than other fibroblast clusters. Similarly, CQ1C^+^CD206^−^ macrophages barely expressed IL6R, and IL6R was only strongly expressed in monocytes and M2 Macrophages (Fig. 8C and D). Previous research has demonstrated that IL-6 and GM-CSF are key signals released by cancer cell-activated CAFs (oral squamous carcinoma-derived fibroblasts) that synergistically induce monocytes to differentiate into M2-like TAMs using a co-culture system [36]. In the field of gastric cancer, researchers have found that elevated IL-6 release at the tumor site may be crucial in encouraging the polarization of M2 macrophages [37]. Follow-up studies further confirmed that IL6 may be secreted by gastric cancer-derived mesenchymal stromal cells [38]. Utilizing the high resolution of single-cell sequencing technology, it was established that only certain fibroblasts secreted IL6. Specifically, LOX^+^ Fibroblasts secreted IL6 under hypoxic conditions, leading to the differentiation of monocytes into M2-like TAM. Several previous studies have demonstrated IL6 expression is upregulated in gastric cancer and affects its progression [39, 40]. Given that M2 Macrophages were likely derived from monocytes, as indicated by the pseudotime analysis, LOX^+^ Fibroblasts may act as a driver to stimulate monocyte differentiation into M2 Macrophages via IL6-IL6R (Fig. 8E).

## Discussion

Previous cancer research has largely overlooked the existence of non-cancerous cells, focusing predominantly on tumor cells. However, cancer is now recognized as being a complex and dynamic ecosystem with constant interactions between cancer cells and the surrounding tumor microenvironment [41]. This has led to a shift in therapeutic approach from a cancer cell-centered approach to one that is TME-centered. The complexity of the TME presents a significant challenge in developing effective anti-cancer therapies, as various factors operating within this intricate ecosystem can block anti-tumor immunity. Immune checkpoints represent one of many tactics tumors implement to evade immune system attacks. Therefore, numerous cutting-edge studies have begun to target other components of the TME, including the tumor inflammatory environment (chemokines, etc.), hypoxic environment, angiogenesis, stromal components (fibroblasts and ECM), and the intrinsic immune system (M2-like macrophage, M1-like macrophage, NK, DC) [42]. Compared to antibodies targeting PD-1/PD-L1 or CTLA-4, clinical evidence for approaches targeting TME is lacking. Many TME studies have relied on preclinical mouse models or small patient cohorts [43, 44]. The lack of understanding of the underlying mechanisms of TME components has hampered the development of new TME-targeting therapies. Therefore, it is urgent for researchers to deeply dissect the crosstalk among the distinct cell types utilizing single-cell multi-omics and deep learning, etc. Here, we observed the intricate nature and heterogeneity of the GC TME and the major cell types’ metabolic properties. Furthermore, two cell subtypes, LOX^+^ Fibroblasts and M2 Macrophages, were enriched in tumor tissue and related to the survival of GC patients. The two cell subpopulations had a strong correlation and were both present in a hypoxic microenvironment. LOX^+^ Fibroblasts may act as a trigger to stimulate monocyte differentiation into M2 Macrophages via IL6-IL6R.

The tumor immune microenvironment (TIME) is significantly influenced by fibroblast heterogeneity. Recently, studies have explained the heterogeneity of fibroblasts to some extent by single-cell analysis. Li et al. discovered a fibroblast subpopulation with high POSTN expression linked to gastric cancer invasion [9]. Similarly, this fibroblast subpopulation was discovered in our study, and it occupied a larger proportion. Of note, we discovered a subtype of fibroblasts called LOX^+^ Fibroblasts that increased during the remodeling of the TME in GC. This subpopulations was related to the GC patients’ prognosis, indicating its participation in the process of GC invasion. LOX is mainly expressed in stromal cells. Recent research has shown that Lysyl oxidase (LOX) is essential for developing and modulating TME. LOX is strongly linked to the hypoxic microenvironment and can promote epithelial-mesenchymal transition via SNAI2 and Twist [45]. Studies have demonstrated that LOX facilitated the interaction of tumor cells with tumor-associated fibroblasts, promoting the invasion of gastric cancer and liver metastases [46]. Li et al. discovered that Lox^+^ fibroblasts play a crucial role in fibrosis in diabetic heart disease [24]. Furthermore, researchers identified LOX expression was significantly increased in recurrent osteosarcoma CAFs compared to primary osteosarcoma, and targeting LOX of CAFs is an effective treatment for recurrent osteosarcoma [47]. According to our research, a fibroblast subpopulation exhibits high levels of LOX expression. Notably, LOX^+^ Fibroblasts were significantly enriched in hypoxia-related pathways, indicating that the tumor’s hypoxic environment interacts with LOX^+^ Fibroblasts. Our study confirmed the existence of this subpopulation and revealed its distinct metabolic state for the first time. Subsequently, the correlation relationship between LOX^+^ Fibroblast and M2 Macrophage was confirmed. Immunofluorescent double-labeling showed the proximity of LOX-positive cells and CD206-positive cells, implying potential crosstalk between these subpopulations. The prognosis of GC patients is impacted by increased infiltration of M2 Macrophage in tumor tissue with immunosuppressive characteristics. M2 Macrophage with immunosuppressive properties was also found in colon cancer liver metastases [48]. Metabolic enrichment analysis revealed that the specific genes of M2 macrophage were enriched in the hypoxic pathway and showed high lipid metabolic activity, indicating that M2 Macrophage underwent metabolic reprogramming in the TME, thereby altering its immune function. We further explored the interaction between LOX^+^ Fibroblast and M2 Macrophage. The synergistic effect of both subpopulations could promote hypoxia, immunomodulation, and epithelial-mesenchymal transition. Meanwhile, patients with high infiltration of both subpopulations had significantly higher immune checkpoint expression than those with low infiltration, implying that the two subpopulations interacted to enhance the formation of an immunosuppressive environment.

Combining pseudotime analysis and receptor-ligand prediction analysis, we predicted that LOX^+^ Fibroblast might promote monocyte differentiation to M2 Macrophage via IL6-IL6R interactions. Interleukin-6 is a multifunctional cytokine that regulates cell growth, differentiation, and function, mediating the response to injury or infection, immune diseases, and cancer [49]. IL-6 is frequently detected in high concentrations in cancer patients’ blood, tissues, and tumor mass. Elevated IL-6 could promote the progression of several tumors, including breast cancer [50], liver cancer [51], and GC [39]. In gastric cancer, IL6 can exert its influence on invasion and progression via the activation of the JAK2/STAT3 pathway, which is the well-known signaling pathway induced by IL6 [52]. It is now understood that IL-6 is secreted by cancer cells, inflammatory cells, and stromal cells. Fibroblast is a major source of IL-6 in the GC TME, and CAF-derived IL-6 activated the JAK1-STAT3 pathway in GC cells through paracrine signaling, leading to the chemotherapy resistance [53]. Prior investigations have verified that IL6 can induce the differentiation of monocytes into M2 macrophages. However, prior research on the origin of IL6 has been insufficient. It is commonly acknowledged that stromal cells or fibroblasts serve as the primary sources of IL6 in GC and other types of malignancies. However, this generalization oversimplifies the complex nature of fibroblasts within the TME. As detailed in the introduction, fibroblasts exhibit high levels of heterogeneity, with differing metabolic characteristics, regulatory mechanisms, and spatial localization within distinct subpopulations. Our research provided more specific information for upcoming TME-targeted therapy by providing hitherto undocumented evidence that LOX^+^ fibroblasts release the majority of CAF-derived IL6. Of note, LOX^+^ fibroblasts, monocytes, and M2 macrophages are in a hypoxic environment, the most common metabolic feature of tumors. The hypoxic environment is known to precisely regulate fibroblasts and immune cells. In colon cancer, under hypoxic conditions, CAF-derived IL-6 activates STAT3 signaling to promote tumor progression or induce drug resistance via a HIF-1a/miR-338-5p/IL-6 feedback loop [54, 55]. Therefore, we proposed the existence of hypoxia– LOX^+^fibroblast–IL6–monocyte– M2 macrophage loop, which induced the progression of gastric cancer and created a specific immunosuppressed state. Disrupting this cycle has the potential to reshape the GC microenvironment. However, it is important to acknowledge that our study had limitations in that it only focused on two specific cell subpopulations within the TME. While these findings shed critical light on the significance of fibroblasts and immune cells, there is still much to learn about the complex interplay of other TME components that may have been overlooked. Our goal is to continue investigating gastric cancer TME with greater precision in future studies. In the future, the in situ expression profiles and spatial distribution of the TME components can be explored with the help of spatial transcriptomics and spatial proteomics. Beyond that, it is more critical to explore the interactions of TME components in more realistic conditions. Three-dimensional tumor-derived organoids better imitate in vivo tissue and are regarded as a more realistic way to studying complicated TME interactions [56]. The prognosis, diagnosis, and development of new therapies for patients will be improved by the integration of these technologies.

In conclusion, LOX^+^fibroblasts produce IL6 in substantial amounts in the hypoxic environment and contribute to the differentiation of monocytes into M2 Macrophage. Meanwhile, LOX^+^fibroblasts interact with M2 Macrophage to promote gastric cancer progression and metastasis, forming a specific immunosuppressive microenvironment. Our study provided detailed information about immune and non-immune cells in the GC TME and emphasized the potential benefit of developing therapeutic approaches that target LOX^+^fibroblast, M2 Macrophage, or their crosstalk molecules.

## Electronic supplementary material

Below is the link to the electronic supplementary material.

Supplementary Material 1

## Data Availability

The data involved in our work are available in the TCGA (https://portal.gdc.cancer.gov/) and GEO (https://www.ncbi.nlm.nih.gov/geo/ ) databases. Codes are deposited in https://github.com/chen12-tmu/GC-scRNAseq.git.

## Abbreviations

GC: gastric cancer
DEG: differentially expressed genes
GSEA: gene set enrichment analysis
GSVA: gene set variation analysis
ICIs: immune checkpoint inhibitors
ITH: intratumoral heterogeneity
QC: quality control
TIME: tumor immune microenvironment
TME: tumor microenvironment
CAF: Cancer-associated fibroblast
GEO: Gene Expression Omnibus
TCGA: The Cancer Genome Atlas
UMAP: uniform manifold approximation and projection
KEGG: Kyoto Encyclopedia of Genes and Genomes
STAD: Stomach adenocarcinoma
eCAF: extracellular matrix CAF
