# Supplementary figures and images for: Revealing the crosstalk between LOX+ fibroblast and M2 macrophage in gastric cancer by single-cell sequencing
Source: BMC Cancer. 2024 Sep 9;24:1117. doi: 10.1186/s12885-024-12861-y (PMC11382413; doi:10.1186/s12885-024-12861-y)

**Supplementary Information**

FigureS1


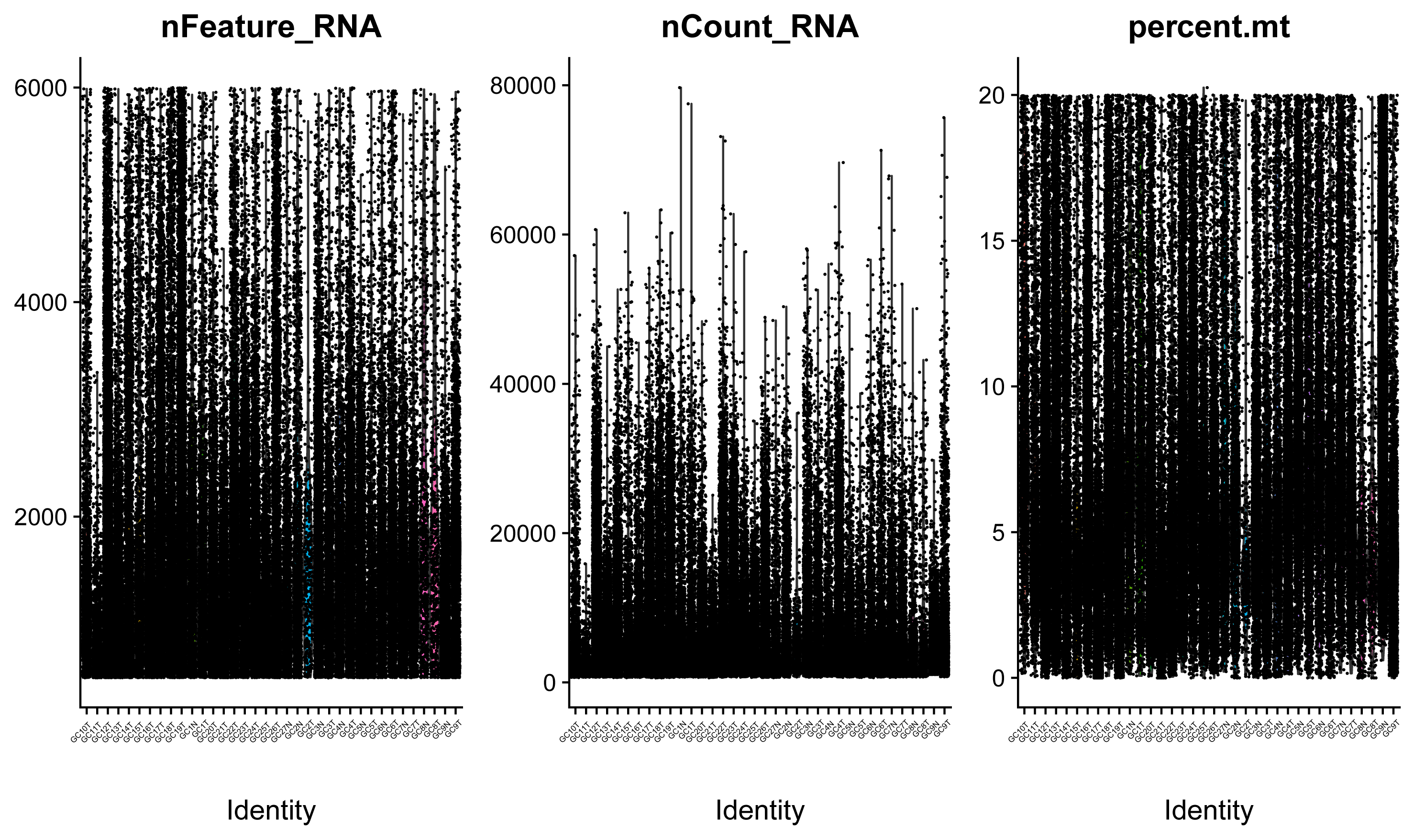


FigureS2


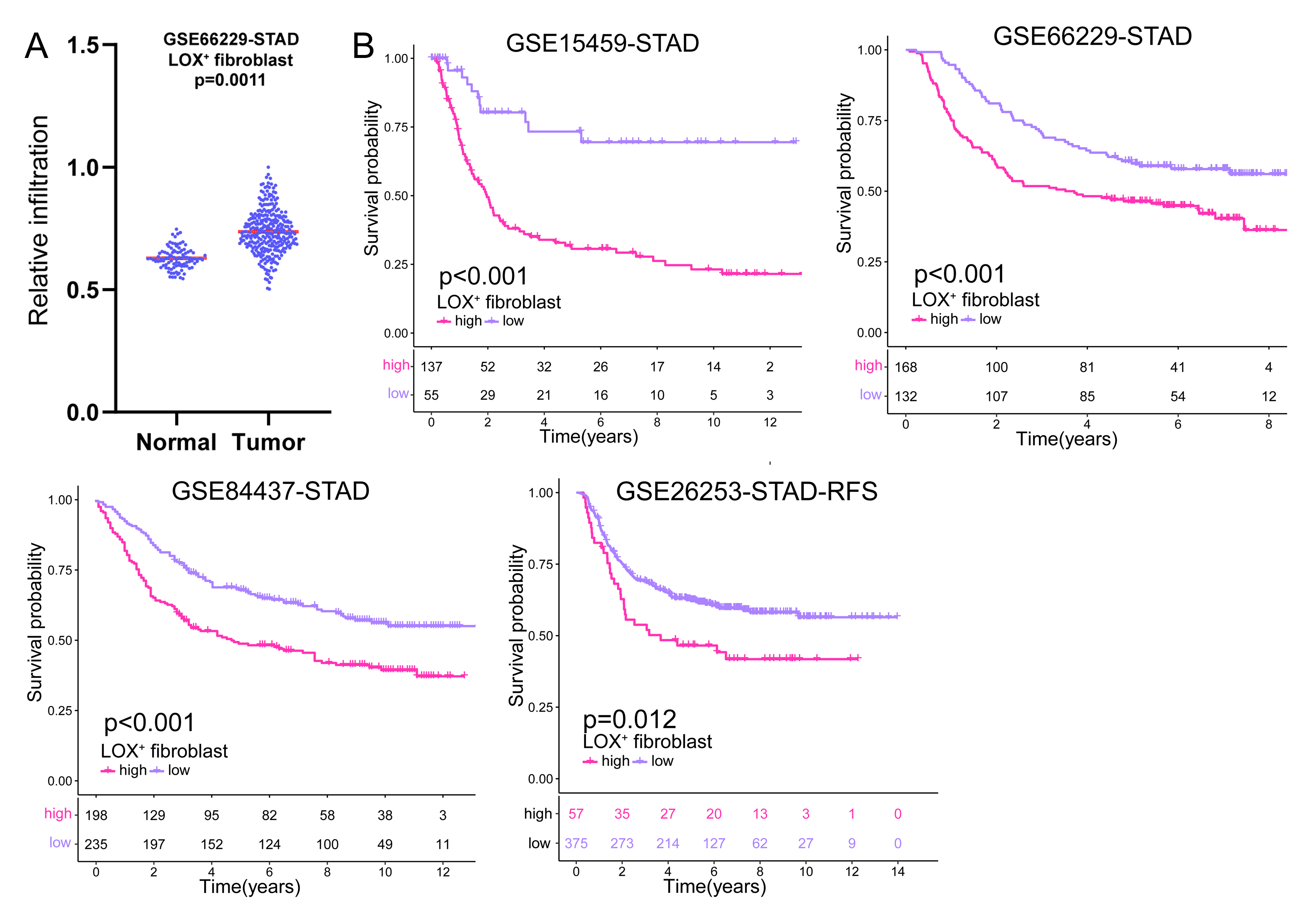

Supplement: Supplementary file 1 — Supplementary Material 1 [file 12885_2024_12861_MOESM1_ESM.docx]
